# Focus on RNA biology

**DOI:** 10.1093/plcell/koad082

**Published:** 2023-03-21

**Authors:** Nancy A Eckardt, Michael J Axtell, Andrea Barta, Xuemei Chen, Brian D Gregory, Hongwei Guo, Pablo A Manavella, Rebecca A Mosher, Blake C Meyers

**Affiliations:** Senior Features Editor, The Plant Cell, American Society of Plant Biologists, USA; Reviewing Editor, The Plant Cell, American Society of Plant Biologists, USA; Department of Biology, The Pennsylvania State University, University Park, PA 16802, USA; Reviewing Editor, The Plant Cell, American Society of Plant Biologists, USA; Max Perutz Labs, Medical University of Vienna, Vienna Biocenter Campus, 1030 Vienna, Austria; Senior Editor, The Plant Cell, American Society of Plant Biologists, USA; Department of Botany and Plant Sciences and Center for Plant Cell Biology, Institute of Integrative Genome Biology, University of California—Riverside, Riverside, CA 92521, USA; Reviewing Editor, The Plant Cell, American Society of Plant Biologists, USA; Department of Biology, University of Pennsylvania, School of Arts and Sciences, Philadelphia, PA 19104, USA; Reviewing Editor, The Plant Cell, American Society of Plant Biologists, USA; Department of Biology, Institute of Plant and Food Science, Southern University of Science and Technology (SUSTech), Shenzhen 518055, China; Guest Editor, The Plant Cell, American Society of Plant Biologists, USA; Instituto de Agrobiotecnología del Litoral (CONICET-UNL), Cátedra de Biología Celular y Molecular, Facultad de Bioquímica y Ciencias Biológicas, Universidad Nacional del Litoral, Santa Fe 3000, Argentina; Guest Editor, The Plant Cell, American Society of Plant Biologists, USA; The School of Plant Sciences, The University of Arizona, Tucson, AZ 85721, USA; Editor-in-Chief, The Plant Cell, American Society of Plant Biologists, USA; Donald Danforth Plant Science Center, St. Louis, MO 63132, USA; Division of Plant Sciences and Technology, University of Missouri-Columbia, Columbia, MO 65211, USA

If DNA is the playbook of life, then RNA, in all its forms, is the company of actors that brings life into being. RNA is likely the original organic molecule that gave rise to DNA as well as enzymes and other proteins that create the flow of genetic information known as the central dogma (DNA -> RNA -> protein). Although the central dogma still holds true generally, our understanding has expanded the concept to encompass reverse transcription of RNA to DNA, regulatory functions of noncoding RNAs, and epigenetic modifications. In this focus issue, we highlight many of the forms and functions of RNA in plants.

The issue includes 10 reviews and 7 research articles. In a vignette-style review by Manavella et al. (2023), 12 experts present and discuss compelling open questions in plant RNA biology and future perspectives in the field. Assmann et al. (2023) review how RNA structure informs function, drawing the intriguing and informative analogy that RNA can be relatively static, like a rock, have catalytic functions of cutting bonds, like scissors, and can adopt myriad functional shapes, like paper. Marquardt et al. (2023) explore the crosstalk between transcription and RNA maturation in plants and its impact on genome integrity and gene expression. Mateo and Staiger (2023) discuss how recent technological achievements have enlarged our understanding of the function and mode of action of plant RNA-binding proteins (RBP) and enabled a genome-wide view of the plant RBPome. Small et al. (2023) take a look at RBPs with respect to RNA processing and maturation in plant organelles (principally chloroplasts and mitochondria). In his “intron-centric” guide to alternative splicing, Petrillo (2023) discusses fundamental aspects of splicing and alternative splicing with a focus on introns. Palos et al. (2023) provide perspectives on long-non-coding (lnc) RNAs, examining key discoveries made in plant lncRNA biology over the past 2 and a half decades as well as challenges and new molecular and computational approaches to lncRNA comparative and functional analyses. Chow and Mosher (2023) review small RNA-mediated DNA methylation during plant reproduction, focusing on the biosynthesis, transport, and function of reproductive short-interfering (si) RNAs. Prall et al. (2023) provide an overview of covalent nucleotide modifications within plant mRNAs, their methods of detection and analysis, and outstanding questions about their function. Heeney and Frank (2023) delve into the knowns and unknowns in the field of long-distance mRNA movement, discussing what defines a functional signaling molecule and how to decipher signals within the inherently noisy data of the mRNA “mobileome.”

In a breakthrough report, Hudzik et al. (2023) report on trans-species microRNAs (miRNAs) in the parasitic plant *Cuscuta campestris*, showing that they have a U6-like snRNA promoter that produces miRNAs with a mechanism distinct from canonical miRNAs, which they speculate may allow these miRNAs to be exported to host plants. Guo et al. (2023) present a large-scale biology study of the translational landscape of bread wheat during grain development, providing new insights into the translational control of gene expression during this critical stage of crop development. Five additional research articles report the discovery of a novel class of small RNAs upregulated by nutrient deprivation in Chlamydomonas (Li et al. 2023), structural insights into the activation of an organellar C-to-U RNA editing enzyme in Arabidopsis (Toma-Fukai et al. 2023), the functional interdependence of subunits of the Arabidopsis m6A methyltransferase complex (Shen 2023), the requirement of DNE1 endoribonuclease for turnover of diverse mRNA substrates in Arabidopsis (Nagarajan et al. 2023), and the synergistic action of the Arabidopsis spliceosome components PRP39a and SmD1b in promoting post-transcriptional transgene silencing (Bazin et al. 2023). The cover of this issue features original artwork by Nicolas Cinquegrani (nicocinque.com).

We encourage authors to continue to submit their best work on plant RNA biology to *The Plant Cell*. Articles published in this area within ~12 mo of this focus issue will be added to an online collection on plant RNA biology, building on the articles presented here.

## References

[koad082-B1] Assmann SM , Chou H-L, Bevilacqua PC. Rock, scissor, paper: how RNA structure informs function. Plant Cell. 2023:35(6):1671–1707. 10.1093/plcell/koad026 36747354 PMC10226581

[koad082-B2] Bazin J , Elvira-Matelot E, Blein T, Jauvion V, Bouteiller N, Cao J, Crespi M, Vaucheret H. Synergistic action of the Arabidopsis spliceosome components PRP39a and SmD1b in promoting post-transcriptional transgene silencing. Plant Cell. 2023:35(6):1917–1935. 10.1093/plcell/koad091 PMC10226559 36970782

[koad082-B3] Chow HT , Mosher RA. Small RNA-mediated DNA methylation during plant reproduction. Plant Cell. 2023:35(6):1787–1800. 10.1093/plcell/koad010 PMC10226566 36651080

[koad082-B4] Guo Y , Chen Y, Wang Y, Wu X, Zhang X, Mao W, Yu H, Guo K, Xu J, Ma L, et al The translational landscape of bread wheat during grain development. Plant Cell. 2023:35(6):1848–1867. 10.1093/plcell/koad075 PMC10226598 36905284

[koad082-B5] Heeney M , Frank MH. The mRNA mobileome: challenges and opportunities for deciphering signals from the noise. Plant Cell. 2023:35(6):1817–1833. 10.1093/plcell/koad063 PMC10226602 36881847

[koad082-B6] Hudzik C , Maguire S, Guan S, Held J, Axtell MJ. *Trans*-species microRNA loci in the parasitic plant *Cuscuta campestris* have a U6-like snRNA promoter. Plant Cell. 2023:35(6):1834–1847. 10.1093/plcell/koad076 PMC10226579 36896651

[koad082-B7] Li Y , Kim E-J, Voshall A, Moriyama EN, Cerutti H. Small RNAs >26 nt in length associate with AGO1 and are upregulated by nutrient deprivation in the alga Chlamydomonas. Plant Cell. 2023:35(6):1868–1887. 10.1093/plcell/koad093 36945744 PMC10226591

[koad082-B8] Manavella PA , Herz MAG, Kornblihtt AR, Sorenson R, Sieburth LE, Nakaminami K, Seki M, Ding Y, Sun Q, Kang H, et al Beyond transcription: compelling open questions in plant RNA biology. Plant Cell. 2023:35(6):1626–1653. 10.1093/plcell/koac346 36477566 PMC10226580

[koad082-B9] Marquardt S , Petrillo E, Manavella PA. Cotranscriptional RNA processing and modification in plants. Plant Cell. 2023:35(6):1654–1670. 10.1093/plcell/koac309 36259932 PMC10226594

[koad082-B10] Mateos JL , Staiger D. Toward a systems view on RNA-binding proteins and associated RNAs in plants: guilt by association. Plant Cell. 2023:35(6):1708–1726. 10.1093/plcell/koac345 36461946 PMC10226577

[koad082-B11] Nagarajan VK , Stuart CJ, DiBattista AT, Accerbi M, Caplan JL, Green PJ. RNA degradome analysis reveals DNE1 endoribonuclease is required for the turnover of diverse mRNA substrates in Arabidopsis. Plant Cell. 2023:35(6):1936–1955. 10.1093/plcell/koad085 37070465 PMC10226599

[koad082-B12] Palos K , Yu L, Railey CE, Nelson Dittrich AC, Nelson ADL. Linking discoveries, mechanisms, and technologies to develop a clearer perspective on plant long noncoding RNAs. Plant Cell. 2023:35(6):1762–1786. 10.1093/plcell/koad027 36738093 PMC10226578

[koad082-B13] Petrillo E . Do not panic: an intron-centric guide to alternative splicing. Plant Cell. 2023:35(6):1752–1761. 10.1093/plcell/koad009 36648241 PMC10226583

[koad082-B14] Prall W , Ganguly DR, Gregory BD. The covalent nucleotide modifications within plant mRNAs: what we know, how we find them, and what should be done in the future. Plant Cell. 2023:35(6):1801–1816. 10.1093/plcell/koad044 36794718 PMC10226571

[koad082-B15] Shen L . Functional interdependence of *N*^6^-methyladenosine methyltransferase complex subunits in Arabidopsis. Plant Cell. 2023:35(6):1901–1916. 10.1093/plcell/koad070 36890720 PMC10226572

[koad082-B16] Small I , Melonek J, Bohne A-V, Nickelsen J, Schmitz-Linneweber C. Plant organellar RNA maturation. Plant Cell. 2023:35(6):1727–1751. 10.1093/plcell/koad049 36807982 PMC10226603

[koad082-B17] Toma-Fukai S , Sawada Y, Maeda A, Shimizu H, Shikanai T, Takenaka M, Shimizu T. Structural insight into the activation of an Arabidopsis organellar C-to-U RNA editing enzyme by active site complementation. Plant Cell. 2023:35(6):1888–1900. 10.1093/plcell/koac318 36342219 PMC10226597

